# Short-term effects of high-dose oral vitamin D3 in critically ill vitamin D deficient patients: a randomized, double-blind, placebo-controlled pilot study

**DOI:** 10.1186/cc10120

**Published:** 2011-03-28

**Authors:** Karin Amrein, Harald Sourij, Gerit Wagner, Alexander Holl, Thomas R Pieber, Karl Heinz Smolle, Tatjana Stojakovic, Christian Schnedl, Harald Dobnig

**Affiliations:** 1 Medical University of Graz, Department of Internal Medicine, Division of Endocrinology and Metabolism, Auenbruggerplatz 15, 8036 Graz, Austria; 2 Medical University of Graz, Department of Internal Medicine, Medical Intensive Care Unit, Auenbruggerplatz 15, 8036 Graz, Austria; 3 Medical University of Graz, Department of Medical Informatics, Statistics and Documentation, Auenbruggerplatz 2, 8036 Graz, Austria; 4 Medical University of Graz, Department of Neurology, Division of Special Neurology, Auenbruggerplatz 22, 8036 Graz, Austria; 5 Medical University of Graz, Clinical Institute of Medical and Chemical Laboratory Diagnostics, Auenbruggerplatz 15, 8036 Graz, Austria

## Abstract

**Introduction:**

Vitamin D deficiency is encountered frequently in critically ill patients and might be harmful. Current nutrition guidelines recommend very low vitamin D doses. The objective of this trial was to evaluate the safety and efficacy of a single oral high-dose vitamin D3 supplementation in an intensive care setting over a one-week observation period.

**Methods:**

This was a randomized, double-blind, placebo-controlled pilot study in a medical ICU at a tertiary care university center in Graz, Austria. Twenty-five patients (mean age 62 ± 16yrs) with vitamin D deficiency [25-hydroxyvitamin D (25(OH)D) ≤20 ng/ml] and an expected stay in the ICU >48 hours were included and randomly received either 540,000 IU (corresponding to 13.5 mg) of cholecalciferol (VITD) dissolved in 45 ml herbal oil or matched placebo (PBO) orally or via feeding tube.

**Results:**

The mean serum 25(OH)D increase in the intervention group was 25 ng/ml (range 1-47 ng/ml). The highest 25(OH)D level reached was 64 ng/ml, while two patients showed a small (7 ng/ml) or no response (1 ng/ml). Hypercalcemia or hypercalciuria did not occur in any patient. From day 0 to day 7, total serum calcium levels increased by 0.10 (PBO) and 0.15 mmol/L (VITD; *P *< 0.05 for both), while ionized calcium levels increased by 0.11 (PBO) and 0.05 mmol/L (VITD; *P *< 0.05 for both). Parathyroid hormone levels decreased by 19 and 28 pg/ml (PBO and VITD, ns) over the seven days, while 1,25(OH)D showed a transient significant increase in the VITD group only.

**Conclusions:**

This pilot study shows that a single oral ultra-high dose of cholecalciferol corrects vitamin D deficiency within 2 days in most patients without causing adverse effects like hypercalcemia or hypercalciuria. Further research is needed to confirm our results and establish whether vitamin D supplementation can affect the clinical outcome of vitamin D deficient critically ill patients.

**EudraCT Number:**

2009-012080-34

**German Clinical Trials Register (DRKS):**

DRKS00000750

## Introduction

Hypocalcemia occurs frequently in patients with critical illness and has been related to adverse clinical outcomes including increased mortality [1-3]. Underlying reasons remain largely unknown but vitamin D deficiency and accompanying secondary hyperparathyroidism may be one of them. However, there is currently no proven intervention that corrects and sustains calcium levels safely and effectively in critical illness. Various forms of calcium and calcitriol supplementation were unable to demonstrate sustained effects and in some cases resulted in hypercalcemia and increased mortality [2]. Several case reports have suggested vitamin D deficiency as a cause of hypocalcemia with adverse outcome, which can be corrected effectively and safely by vitamin D supplementation [4,5].

Published data [6-9] as well as our own observations suggest that most critically ill patients are vitamin D deficient, although there are concerns in this special group of patients that acute fluid shifts and inflammatory states may influence the assessment and interpretation of vitamin status [10-13]. There is now extensive literature on adverse health outcomes in patients with vitamin D deficiency reporting increased mortality, cardiovascular events and impaired function of the immune and musculoskeletal systems [14,15]. Many tissues, including cardiomyocytes and immune cells have a nuclear vitamin D receptor and respond to 1,25-dihydroxyvitamin D (1,25(OH)2D) [16,17]. This active form of vitamin D is either derived from the kidney where it is secreted into the circulation or is formed within the target cell by hydroxylation of 25-hydroxyvitamin D (25(OH)D) to 1,25(OH)2D. A large potential for beneficial clinical effects of a normalized vitamin D status in the setting of critically ill patients has recently been brought forward and attracted attention [18]. The beneficial effects of vitamin D on muscle, heart and immune function could be of particular interest in critical care, especially in patients with respiratory failure or those who are on mechanical ventilation for other reasons.

The current recommendation for parenteral vitamin D3 is 200 IU per day [19], a dose that is too low to have an effect on serum vitamin D levels in patients with established vitamin D deficiency. European Society for ClinicalNutrition and Metabolism (ESPEN) guidelines do not specifically address vitamin D supplementation [20,21].

Uncertainty exists on the dosage of vitamin D supplementation that should be used. Vitamin D intoxication can potentially be life-threatening but the majority of officially recorded cases could be related to prolonged intakes of more than 40,000 IU per day [22]. Two study groups have recently shown safety and efficacy of a single high-dose cholecalciferol administration in rheumatologic and elderly patients [23,24], and mean increases of 26 ng/ml at a dose of 300,000 IU [24] and 28 ng/ml at 500,000 IU [23] one to three months after administration were reported. The feasibility of 60,000 IU cholecalciferol given twice a week to critically ill patients has been studied by a Spanish group but in this report safety issues were not addressed [25]. Except for dosing problems, further main limitations of vitamin D administration include the contraindication for intramuscular injections in most ICU patients, the unavailability of parenteral high-dose vitamin D formulations and finally unreliable gastrointestinal absorption after oral dosing due to multifactorial gastrointestinal dysfunction, changes in gastrointestinal blood supply and motility, as well as the presence of sepsis and multiorgan failure.

Our hypothesis was that a single ultra-high dose may overcome some of these limitations and is able to restore normal 25(OH)D levels without causing adverse effects. The results obtained from this study should provide a basis of an easy-to-administer dosing regimen for other prospective intervention trials in an ICU setting.

## Materials and methods

The trial was started in August 2009 and terminated in January 2010. In a single-center setting of a tertiary care university hospital, 25(OH)D-deficient adult patients (levels ≤20 ng/ml) with an expected stay in the medical ICU of more than 48 hours were randomly assigned to receive either 540,000 IU of vitamin D3 (which equalled three bottles of a commercially available vitamin D preparation in Austria: Oleovit D3^® ^solution; 400 IU vitamin D3/drop) dissolved in 45 ml of herbal oil ("VITD" group) or herbal oil only (placebo group, "PBO" group). The study medication was prepared, labelled and randomized by a pharmacist and physician not involved in the trial. The randomization was performed with sealed envelopes and all aspects of the trial were performed in a double-blind fashion. Exclusion criteria included moribund patients expected to die within 24 hours, hypercalcemia, ileus, pregnancy, and a history of kidney stones, sarcoidosis or tuberculosis. Most patients received the study medication via a nasogastric feeding tube, while two patients in each group were able to take it orally.

The institutional ethical committee approved the trial before commencement (protocol number: 20-366 ex 08/09; EudraCT 2009-012080-34, DRKS00000750). Patients able to follow study protocol gave informed consent in written form, while mechanically ventilated patients were asked for their approval after regaining consciousness. 25(OH)D was measured by ELISA (Immunodiagnostic Systems, Boldon, UK). On weekdays, 25(OH)D analyses in our institution were performed on a daily basis. 1,25-(OH)2D was analysed with ELISA (Immunodiagnostic Systems, Boldon, UK). Serum electrolytes as well as urinary creatinine and electrolytes were measured on a Roche Modular (Roche Diagnostics, Mannheim, Germany). Parathyroid hormone (PTH) was analyzed by electrochemiluminescence immunoassay (Elecsys 2010, Roche Diagnostics, Mannheim, Germany). Serum triglycerides were determined enzymatically with the GPO-PAP method (Roche Diagnostics, Mannheim, Germany). N-terminal prohormone brain natriuretic peptide (NT-proBNP) was measured by electrochemiluminescence (Elecsys 2010, Roche Diagnostics, Mannheim, Germany).

The active study participation for each patient lasted seven days with an optional follow up at 28 days in case patients were still residing within the hospital. The primary endpoint was the percentage of patients who reached 25(OH)D levels of 30 ng/ml or above on day 7. Secondary endpoints included various biochemical and clinical parameters related to vitamin D and mineral metabolism (i.e. 1,25(OH)2D, serum PTH levels, serum electrolytes, urinary calcium/creatinine ratio, and NT-proBNP levels), length of hospital and ICU stay, duration of mechanical ventilation and catecholamine support as well as hospital mortality.

### Statistical analysis

Data were analyzed using SPSS 16.0 (SPSS Inc., Chicago, IL, USA) and are expressed as mean ± standard deviation or median (interquartile range) as appropriate. Between-group comparisons were carried out using Mann-Whitney-U test and chi squared-test where appropriate. Unifactorial repeated measures analysis of variance was used to analyze changes in blood and urinary parameters.

## Results

Out of 25 patients enrolled we could obtain results from 20 at day 7 (10 in each group). The 25(OH)D level in the VITD group was 13.1 ± 2.0 ng/ml (mean ± standard deviation) on day 0, 35.1 ± 15.2 ng/ml on day 3 and 38.2 ± 16.5 ng/ml on day 7 (respective interquartile ranges 3.5, 18.5, and 21.5), while in the PBO group it was 14.1 ± 3.7 ng/ml on day 0, 14.5 ± 4.6 ng/ml on day 3 and 13.7 ± 4.2 ng/ml on day 7 (respective interquartile ranges 7.2, 6.2, and 7.5).

**Figure 1:**
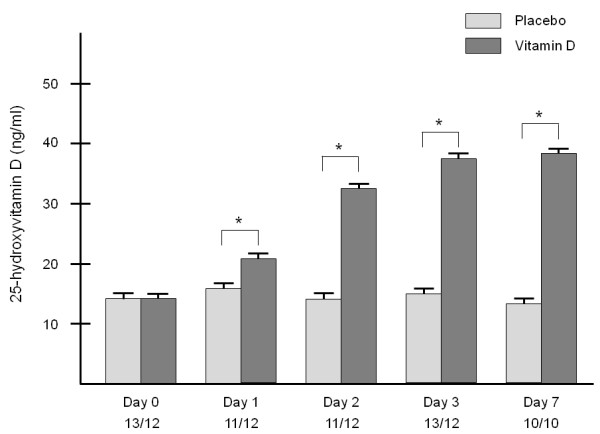
**Mean 25-hydroxyvitamin D levels (±SEM) after a single enteral dose of 540,000 IU vitamin D3 or placebo in vitamin D deficient patients at a medical ICU**. The numbers given below the study days refer to the number of patients per group.

Clinical characteristics of the participants are given in Table 1. Biochemical changes during the follow-up period are shown in Table 2. Already on day 1, 25(OH)D was significantly higher in the intervention group (Figure 1). Eight out of 10 patients showed a normalization of 25(OH)D levels (≥30 ng/ml), and the highest level achieved was 64 ng/ml on day 3.

**Table 1:** Clinical characteristics at baseline and secondary clinical outcomes of the two groups

	Placebo (*n *= 13)	Vitamin D (*n *= 12)	*P *value
***Baseline characteristics***			
Male - n (%)	10 (77)	9 (75)	0.91
Age (years)	64.1 ± 16.4	61.1 ± 16.7	0.65
Body mass Index (kg/m^2^)	29.3 ± 8.5	28.7 ± 9.7	0.87
SAPS II (ICU admission)	38.3 ± 22.3	33.2 ± 12.2	0.47
TISS (study start)	38.0 ± 7.2	30.2 ± 7.1	0.08
Renal replacement therapy - n (%)	3 (23)	1 (8)	0.31
Diagnostic category	infectious (4)	infectious (6)	
	cardiovascular (4)	cardiovascular (3)	
	neurologic (4)	neurologic (1)	-
	gastrointestinal (1)	gastrointestinal (0)	
	other (0)	other (2)	
Mechanical ventilation - n (%)	11 (85)	10 (83)	0.93
Vasopressor therapy - n (%)	12 (92)	8 (67)	0.10
***Outcome variables***			
Mechanical ventilation - duration (hours)	163 (91-541)	229 (82-366)	0.88
Vasopressor therapy - duration (hours)	146 (48-351)	65 (42-299)	0.56
Hospital stay, days (from study start)	15 (8-38)	16 (8-32)	0.97
ICU stay, days (from study start)	6 (3-23)	10 (5-21)	0.54
Hospital mortality - n (%)	6 (46)	6 (50)	0.84

Continuous data are presented as means (standard deviation) or median (interquartile range), categorical data are shown in total numbers (percentages).

SAPS, simplified acute physiology score; TISS 28, Therapeutic Intervention Scoring System.

**Table 2:** Biochemical changes during the seven-day observational period

	Day 0	Day 1	Day 2	Day 3	Day 7
25(OH)D (ng/ml)					
*Placebo group*	14.1	15.3*	15.0*	14.5*	13.7*
*Vitamin D group*	13.1	20.5*^§^	33.1*^§^	35.1*^§^	38.2*^§^
1,25(OH)_2_D (pmol/l)					
*Placebo group*	50.6	36.3*	36.6	47.1	67.0
*Vitamin D group*	68.6	89.5*^§^	175.2*^§^	170.8*^§^	114.2
PTH (pg/ml)					
*Placebo group*	91.3	72.0	79.0	75.7	71.7
*Vitamin D group*	73.7	65.1	77.3	100.4	52.0
Ionized serum calcium (mmol/l)					
*Placebo group*	1.03	^-^	-	1.05	1.14^§^
*Vitamin D group*	1.09	^-^	-	1.11	1.14^§^
Total serum calcium (mmol/l)					
*Placebo group*	2.03	2.02	1.98	2.02	2.13^§^
*Vitamin D group*	2.05	2.05	2.09	2.12	2.20^§^
Serum phosphorus (mmol/l)					
*Placebo group*	1.57*	1.34	1.49*	1.51*	1.22
*Vitamin D group*	1.01*	1.20	1.08*	1.07*	1.17
Triglycerides (mg/dl)					
*Placebo group*	111	126	-	-	116
*Vitamin D group*	180	175	-	-	178
NTproBNP (pg/ml)					
*Placebo group*	2100	-	-	-	2120
*Vitamin D group*	3089	-	-	-	3117
Urinary calcium/creatinine ratio (mmol/mmol)					
*Placebo group*	0.38	0.24	0.14	0.15	0.41
*Vitamin D group*	0.38	0.19	0.27	0.28	0.27

1,25(OH)_2_D, 1,25-dihydroxyvitamin D; 25(OH)D, 25-hydroxyvitamin D, NTproBNP, N-terminal prohormone brain natriuretic peptide; PTH, parathyroid hormone.

§, *P *< 0.05 for intra-group comparisons (calculated in relation to baseline values) and *, *P *< 0.05 for between-group comparisons.

Two patients in the VITD group demonstrated either a small (7 ng/ml) or no (1 ng/ml) increase in 25(OH)D levels. In one patient, this may be attributable to gastrointestinal dysfunction after hypoxia related to near-drowning and resuscitation, and in the other to a history of sclerodermiform gastrointestinal graft-versus-host disease after allogeneic stem cell transplantation. Individual patient data are given in Table 3.

**Table 3:** Clinical characteristics of individuals in the VITD group

									25(OH)D levels		
	Gender	Age	BMI	Diagnosis	Gastrointestinal function	Application	VP	MV	Day 0	Day 3	Day 7
02	female	83	33.2	Pneumonia	normal	FT	yes	yes	13.7	64.2	59.4
05	male	72	30.1	Heart failure, renal transplant	normal	FT	yes	yes	12.2	33.1	34.8
06	male	77	21.1	Myelodysplastic syndrome, sepsis	normal	O	no	no	10.1	36.5	-
08	male	84	23.1	Pneumonia	normal	FT	yes	yes	16.0	50.6	63.4
11	male	55	36.6	Wegener's granulomatosis, renal failure	normal	FT	no	yes	10.8	26.9	29.8
14	female	45	21.7	CPR (near-drowning)	hypoxic gastrointestinal dysfunction	FT	yes	yes	9.7	8.3	10.3
15	male	78	27.0	Seizures	normal	FT	no	yes	10.1	63.5	-
16	male	58	27.4	H1N1 influenza, renal transplant	normal	O	no	no	13.7	30.5	50.3
18	male	47	27.5	H1N1 influenza, renal transplant	normal	FT	yes	yes	15.1	42.9	29.3
22	female	51	24.8	CPR (ventricular fibrillation)	normal	FT	yes	yes	12.8	36.6	43.6
25	male	46	17.3	Allogeneic stem cell transplantation for chronic myeloid leukemia	sclerodermiform gastrointestinal graft-versus-host disease	FT	yes	yes	15.4	24.5	22.1
26	male	37	54.6	Pneumonia	normal	FT	yes	yes	12.0	33.0	38.8

25(OH)D, 25-hydroxyvitamin D, BMI, body mass index; CPR, cardiopulmonary resuscitation; FT, feeding tube; MV, mechanical ventilation; O, oral; VITD, vitamin D; VP, vasopressor.

Total serum calcium and ionized calcium increased significantly in both groups, whereas 1,25(OH)2D levels did so in the VITD group only. The proportion of patients with normocalcemia on day 3 and 7 (ionized calcium ≥1.15 mmol/l) was not significantly different between both groups. Serum PTH levels had declined although not significantly in both groups at day 7. In this pilot study there were no differences in clinical outcome variables such as duration of mechanical ventilation, vasopressor dependency, or mortality between the two groups (Table 1). Interestingly, hospital mortality in patients who became normocalcemic on day 3 (ionized calcium ≥1.15 mmol/l) compared with those who remained in the hypocalcemic range was lower (20% versus 55%) although this difference did not reach statistical significance.

## Discussion

In this pilot study an enteral ultra-high dose of 540,000 IU of vitamin D3 given once to vitamin D-deficient patients was able to rapidly normalize 25(OH)D levels in most patients in a medical ICU setting. Conversely, routine replacement with daily doses of 200 IU of cholecalciferol in the PBO group did not have any effect on 25(OH)D levels. The highest achieved 25(OH)D level in the VITD group was 64 ng/ml and far away from levels thought to be indicative of toxicity (>200 ng/ml) [22]. However, the individual maximal rise following application of this single dose of vitamin D3 was variable, ranging from 1 to 47 ng/ml. In other (non-critically ill) populations the effect of a large dose of vitamin D yielded somewhat comparable or slightly higher 25(OH)D levels one week after administration [24,26].

The progressive increase in 25(OH)D levels may not only be explained by initial differences in intestinal absorption of vitamin D3, but also by limited capacity of the vitamin D binding protein transport system, or that of the liver to hydroxylate the precursor. Moreover, there may be interindividual differences applying to storage effects, whereby cholecalciferol is taken up or released by adipose and muscle tissues. However, most of the vitamin D that enters the body is catabolized and excreted without ever being stored in tissues, and without ever becoming 25(OH)D [27]. We thus cannot exclude that either a change towards smaller dosages or more frequent dosing intervals may also have yielded the desired effects and normalized 25(OH)D levels.

Our study provides novel information on how vitamin D replacement may correct vitamin D deficiency. Of interest in this study is also the plasma kinetics of 1,25(OH)2D that in this small patient sample indicated progressive and at day 3 almost three-fold increase above baseline levels that seemed to rapidly decline thereafter. As serum PTH and total calcium levels, which both modulate CYP27B1 activity, remained unchanged during that period, it seems that this transient rise in 1,25(OH)2D was mainly attributable to the boost of suddenly available 25(OH)D substrate. A plausible reason for the subsequent decrease in 1,25(OH)2D levels following day 3 may be the induction of CYP24 hydroxylase gene that shifts 25(OH)D more towards the production of inactive 24,25(OH)2D thus limiting the rise of 1,25(OH)2D levels. Serum calcium levels increased in both groups over the one-week observation period (without a respective change in serum protein) and likely represent the consequence of acute immobilization on bone turnover dynamics. It is of note that despite the marked increases in serum 25(OH)D and 1,25(OH)2D as well as serum calcium levels there was no clear-cut suppression of PTH secretion in the VITD group and only a tendency for lower levels. We speculate that these data may be indicative of a change in calcium responsiveness of the parathyroid gland in critically ill patients. Hypercalcemia was not encountered at any time-point in this patient setting. Despite the high fat content of the study medication, triglyceride levels did not change significantly in either group, neither did NTproBNP serum levels, as opposed to recent results in older patients with heart failure [28].

The most important limitation of this pilot study is its size. The results may not be applicable to every patient group encountered in the ICU. Not surprisingly, we did not find any differences in clinical outcome parameters; however, the sample size and its statistical power did not allow for such analysis. A larger trial addressing these issues is currently being performed at our institution.

## Conclusions

The administration of high-dose vitamin D continues to be a promising and inexpensive intervention in vitamin D-deficient critically ill patients that has a low-risk profile and a broad therapeutic window. Further trials are needed to confirm that the dosing scheme proposed by the results of this study is indeed reasonable and most importantly, that normalization of 25(OH)D status is also related to improvement in clinical outcome parameters.

## Key messages

• Vitamin D deficiency is highly prevalent and is linked to increased morbidity and mortality in the general population.

• Vitamin D plays a crucial role in calcium homeostasis besides other skeletal and non-skeletal effects; it has an excellent safety profile and likely a broad therapeutic window.

• The scarce available data suggest that vitamin D deficiency affects the majority of patients in the ICU despite vitamin D supplementation in enteral and parenteral nutrition.

• This pilot study is the first randomized controlled trial that investigates short-term effects of high-dose vitamin D in a limited number of critically ill patients.

• Further research is needed to study whether vitamin D deficiency is an independent risk factor for critically ill patients and if correction leads to clinical benefits.

## Abbreviations

25(OH)D: 25-hydroxyvitamin D; 1,25(OH)2D: 1,25-dihydroxyvitamin D; ELISA: enzyme-linked immunosorbent assay; NTproBNP: N-terminal prohormone brain natriuretic peptide; PBO: placebo; PTH: parathyroid hormone; VITD: vitamin D.

## Competing interests

The authors declare that they have no competing interests.

## Authors' contributions

KA and HD designed the study, performed the statistical analysis and drafted the manuscript. AH, HS and CS participated in data collection, analysis and preparation of the manuscript. GW participated in data collection and analysis. TP, KS and TS revised the manuscript.

## References

[B1] Carlstedt F Lind L Rastad J Stjernstrom H Wide L Ljunghall S Parathyroid hormone and ionized calcium levels are related to the severity of illness and survival in critically ill patients Eur J Clin Invest 1998 28 898 903 10.1046/j.1365-2362.1998.00391.x 9824432

[B2] Forsythe RM Wessel CB Billiar TR Angus DC Rosengart MR Parenteral calcium for intensive care unit patients Cochrane Database Syst Rev 2008 8 CD006163 10.1002/14651858.CD006163.pub2 PMC13716406 18843706

[B3] Hastbacka J Pettila V Prevalence and predictive value of ionized hypocalcemia among critically ill patients Acta Anaesthesiol Scand 2003 47 1264 1269 10.1046/j.1399-6576.2003.00236.x 14616325

[B4] Lee P Milliken S Center JR Hypocalcaemic cardiac failure post BMT secondary to unrecognized vitamin D deficiency Bone Marrow Transplant 2008 42 363 364 10.1038/bmt.2008.178 18574443

[B5] Lee P Samaras K Glanville AR Center JR Transplant recipients on the edge of the hypocalcemia abyss J Heart Lung Transplant 2009 28 93 95 10.1016/j.healun.2008.09.010 19134537

[B6] Lee P Eisman JA Center JR Vitamin D deficiency in critically ill patients N Engl J Med 2009 360 1912 1914 10.1056/NEJMc0809996 19403914

[B7] Van den Berghe G Van Roosbroeck D Vanhove P Wouters PJ De Pourcq L Bouillon R Bone turnover in prolonged critical illness: effect of vitamin D J Clin Endocrinol Metab 2003 88 4623 4632 10.1210/jc.2003-030358 14557432

[B8] Lucidarme O Messai E Mazzoni T Arcade M du Cheyron D Incidence and risk factors of vitamin D deficiency in critically ill patients: results from a prospective observational study Intensive Care Med 2010 20373095 10.1007/s00134-010-1875-8

[B9] Jeng L Yamshchikov AV Judd SE Blumberg HM Martin GS Ziegler TR Tangpricha V Alterations in vitamin D status and anti-microbial peptide levels in patients in the intensive care unit with sepsis J Transl Med 2009 7 28 10.1186/1479-5876-7-28 19389235 PMC2684740

[B10] Krishnan A Ochola J Mundy J Jones M Kruger P Duncan E Venkatesh B Acute fluid shifts influence the assessment of serum vitamin D status in critically ill patients Crit Care 2009 14 R216 10.1186/cc9341 PMC3219984 21110839

[B11] Louw JA Werbeck A Louw ME Kotze TJ Cooper R Labadarios D Blood vitamin concentrations during the acute-phase response Crit Care Med 1992 20 934 941 10.1097/00003246-199207000-00007 1617986

[B12] Vasilaki AT Leivaditi D Talwar D Kinsella J Duncan A O'Reilly DS McMillan DC Assessment of vitamin E status in patients with systemic inflammatory response syndrome: plasma, plasma corrected for lipids or red blood cell measurements? Clin Chim Acta 2009 409 41 45 10.1016/j.cca.2009.08.008 19698706

[B13] Galloway P McMillan DC Sattar N Effect of the inflammatory response on trace element and vitamin status Ann Clin Biochem 2000 37 289 297 10.1258/0004563001899429 10817241

[B14] Holick MF Vitamin D deficiency N Engl J Med 2007 357 266 281 10.1056/NEJMra070553 17634462

[B15] Dobnig H Pilz S Scharnagl H Renner W Seelhorst U Wellnitz B Kinkeldei J Boehm BO Weihrauch G Maerz W Independent association of low serum 25-hydroxyvitamin d and 1,25-dihydroxyvitamin d levels with all-cause and cardiovascular mortality Arch Intern Med 168 1340 1349 10.1001/archinte.168.12.1340 18574092

[B16] Bouillon R Carmeliet G Verlinden L van Etten E Verstuyf A Luderer HF Lieben L Mathieu C Demay M Vitamin D and human health: lessons from vitamin D receptor null mice Endocr Rev 2008 29 726 776 10.1210/er.2008-0004 18694980 PMC2583388

[B17] Verstuyf A Carmeliet G Bouillon R Mathieu C Vitamin D: a pleiotropic hormone Kidney Int 2010 78 140 145 10.1038/ki.2010.17 20182414

[B18] Lee P Nair P Eisman JA Center JR Vitamin D deficiency in the intensive care unit: an invisible accomplice to morbidity and mortality? Intensive Care Med 2009 35 2028 2032 10.1007/s00134-009-1642-x 19756497

[B19] Kelly DG Guidelines and available products for parenteral vitamins and trace elements JPEN J Parenter Enteral Nutr 2002 26 S34 36 10.1177/014860710202600510 12216718

[B20] Kreymann KG Berger MM Deutz NE Hiesmayr M Jolliet P Kazandjiev G Nitenberg G van den Berghe G Wernerman J Ebner C Hartl W Heymann C Spies C ESPEN Guidelines on Enteral Nutrition: intensive care Clin Nutr 2006 25 210 223 10.1016/j.clnu.2006.01.021 16697087

[B21] Singer P Berger MM Van den Berghe G Biolo G Calder P Forbes A Griffiths R Kreyman G Leverve X Pichard C Espen ESPEN Guidelines on Parenteral Nutrition: intensive care Clin Nutr 2009 28 387 400 10.1016/j.clnu.2009.04.024 19505748

[B22] Vieth R Critique of the considerations for establishing the tolerable upper intake level for vitamin D: critical need for revision upwards J Nutr 2006 136 1117 1122 16549491 10.1093/jn/136.4.1117

[B23] Bacon CJ Gamble GD Horne AM Scott MA Reid IRL High-dose oral vitamin D3 supplementation in the elderly Osteoporos Int 2009 20 1407 1415 10.1007/s00198-008-0814-9 19101755

[B24] von Restorff C Bischoff-Ferrari HA Theiler R High-dose oral vitamin D3 supplementation in rheumatology patients with severe vitamin D3 deficiency Bone 2009 45 747 749 10.1016/j.bone.2009.06.012 19539796

[B25] Mata-Granados JM Vargas-Vasserot J Ferreiro-Vera C Luque de Castro MD Pavon RG Quesada Gomez JM Evaluation of vitamin D endocrine system (VDES) status and response to treatment of patients in intensive care units (ICUs) using an on-line SPE-LC-MS/MS method J Steroid Biochem Mol Biol 2010 20399267 10.1016/j.jsbmb.2010.03.078

[B26] Whyte MP Haddad JG Jr Walters DD Stamp TC Vitamin D bioavailability: serum 25-hydroxyvitamin D levels in man after oral, subcutaneous, intramuscular, and intravenous vitamin D administration J Clin Endocrinol Metab 1979 48 906 911 10.1210/jcem-48-6-906 447796

[B27] Lawson DE Sedrani SH Douglas J Interrelationships in rats of tissue pools of cholecalciferol and 25-hydroxycholecalciferol formed in u.v. light Biochem J 1986 233 535 540 3006671 10.1042/bj2330535 PMC1153058

[B28] Witham MD Crighton LJ Gillespie ND Struthers AD McMurdo ME The effects of vitamin D supplementation on physical function and quality of life in older patients with heart failure: a randomized controlled trial Circ Heart Fail 2010 3 195 201 10.1161/CIRCHEARTFAILURE.109.907899 20103775

